# Type I Interferon: Potential Therapeutic Target for Psoriasis?

**DOI:** 10.1371/journal.pone.0002737

**Published:** 2008-07-16

**Authors:** Yihong Yao, Laura Richman, Chris Morehouse, Melissa de los Reyes, Brandon W. Higgs, Anmarie Boutrin, Barbara White, Anthony Coyle, James Krueger, Peter A. Kiener, Bahija Jallal

**Affiliations:** 1 MedImmune, Inc., Gaithersburg, Maryland, United States of America; 2 Center for Clinical and Translational Science, Rockefeller University, New York, New York, United States of America; University College Dublin, Ireland

## Abstract

**Background:**

Psoriasis is an immune-mediated disease characterized by aberrant epidermal differentiation, surface scale formation, and marked cutaneous inflammation. To better understand the pathogenesis of this disease and identify potential mediators, we used whole genome array analysis to profile paired lesional and nonlesional psoriatic skin and skin from healthy donors.

**Methodology/Principal Findings:**

We observed robust overexpression of type I interferon (IFN)–inducible genes and genomic signatures that indicate T cell and dendritic cell infiltration in lesional skin. Up-regulation of mRNAs for IFN-α subtypes was observed in lesional skin compared with nonlesional skin. Enrichment of mature dendritic cells and 2 type I IFN–inducible proteins, STAT1 and ISG15, were observed in the majority of lesional skin biopsies. Concordant overexpression of IFN-γ and TNF-α–inducible gene signatures occurred at the same disease sites.

**Conclusions/Significance:**

Up-regulation of TNF-α and elevation of the TNF-α–inducible gene signature in lesional skin underscore the importance of this cytokine in psoriasis; these data describe a molecular basis for the therapeutic activity of anti–TNF-α agents. Furthermore, these findings implicate type I IFNs in the pathogenesis of psoriasis. Consistent and significant up-regulation of type I IFNs and their associated gene signatures in psoriatic skin suggest that type I IFNs may be potential therapeutic targets in psoriasis treatment.

## Introduction

Psoriasis is a debilitating skin disease that affects approximately 2% to 3% of the US population. Although originally viewed as a disease primarily caused by dysfunction of keratinocytes, psoriasis is now recognized as a complicated immune system disorder that involves the interplay of innate immunity and adaptive immune response [1]–[3]. Epidermal hyperplasia; aberrant keratinocyte proliferation and differentiation; angiogenesis; infiltration of T lymphocytes; dendritic cells and neutrophils; and elements of innate immunity are all thought to contribute to the pathogenesis of the disease [4], [5]. Currently, the biologics approved by the US Food and Drug Administration to treat psoriasis target either T cells or TNF-α. Alefacept (Amevive®, Astellas Pharma US, Inc.) inhibits CD2 cell binding to lymphocyte function–associated antigen 3 (LFA-3). Efalizumab (Raptiva®, Genentech, Inc., South San Francisco, CA) binds to the CD11a subunit of LFA-1, thereby preventing LFA-1 from binding to intercellular adhesion molecule 1. Etanercept (Enbrel®, Immunex Corporation, Thousand Oaks, CA), infliximab (Remicade®, Centocor, Inc., Malvern, PA), and adalimumab (Humira®, Abbott Laboratories, North Chicago, IL) are all TNF-α blockers and have clinical response rates that range from 25% to 50% in psoriatic patients. Despite the success of anti–TNF-α therapies, the involvement of TNF-α in disease pathogenesis is not yet fully understood.

The limited clinical response associated with some currently available therapeutics and uncertainties regarding the complexity of psoriasis raise the possibility of other factors being implicated in the pathogenesis. IL-12/IL-23 has been shown to be involved in psoriasis. IL-23 is a key inducer of IFN-γ synthesis in T cells. The activation of IFN-γ signaling in lesional skin of psoriatic patients has been confirmed by genomic profiling [6]. Increased expression of mRNAs encoding p19 and p40 subunits of IL-23 has been found in lesional skin of psoriatic patients [7]. In clinical trials of psoriasis treatments, targeting of IL-12/IL-23 has resulted in significant clinical benefits for patients. Administration of an anti–p40 antibody to a small number of psoriatic patients showed significantly improved clinical activity [8].

Recently, Mee et al showed that IL-1β was constitutively expressed by keratinocytes in vivo and overexpressed in lesional skin [9]. Boniface et al demonstrated that oncostatin M (OSM), a member of the IL-6 family of cytokines, was a potent keratinocyte activator similar to TNF-α, IL-1, IL-17, and IL-22. They also showed that OSM was associated with cutaneous inflammatory responses [10]. IL-22, an effector cytokine mainly produced by T cells, and IL-17 were found to mediate cross talk between the immune system and epithelial cells and might have essential functions in host defense and the pathogenesis of psoriasis [11]. It remains to be seen whether drugs directed at these potential therapeutic targets translate to meaningful clinical response rates in psoriasis patients.

Given the limited benefit and lack of mechanistic understanding of currently approved TNF-α blockers, as well as the preliminary nature of the studies of additional molecules, we wanted to identify other factors that might contribute to the pathogenesis of disease. Such factors could represent additional therapeutic opportunities. For the first time, we used the Affymetrix® whole genome U133 plus v2.0 array platform to profile a large panel of paired lesional and nonlesional skin biopsies from psoriatic patients and to compare the skin profiles from sex- and age-matched healthy donors.

## Results

### The IFNα/β signaling pathway is significantly activated in lesional skin of psoriatic patients

To identify characteristics of cutaneous alterations specific to psoriasis, we used an Affymetrix human genome U133 plus v2.0 array platform to profile skin samples from donors classified as normal (n = 21), 26 paired nonlesional and lesional skin biopsies from 24 psoriatic patients, and lesional skin biopsies from an additional 5 psoriatic patients (the paired nonlesional skin either did not yield sufficient cRNA for hybridization, or scanned arrays had high scaling factors that were more than 3-fold above the average). Probe-level summaries were calculated using the GC-RMA normalization algorithm in Stratagene's ArrayAssist® Lite software package. Significance analysis of microarrays with control of the false discovery rate (see Materials and Methods) was used to select differentially regulated genes in psoriasis (pairwise comparison among lesional and nonlesional skin, lesional skin and skin from healthy donors, and nonlesional and skin from healthy donors). Probe sets with ≥2-fold change and *q* value≤0.05 were considered to be differentially regulated.

Overall, we observed that 1408 probe sets were up-regulated and 1465 probe sets were down-regulated in lesional skin compared with nonlesional skin. Although the number of down-regulated genes outnumbered the up-regulated genes in lesional skin, the up-regulated genes generally showed a much larger magnitude of differential regulation overall. For example, 318 probe sets were up-regulated ≥4-fold in lesional skin, whereas only 84 probe sets were down-regulated by ≥4-fold in lesional skin. Subsequently, our analysis focused mostly on the up-regulated genes.

**Figure 1:**
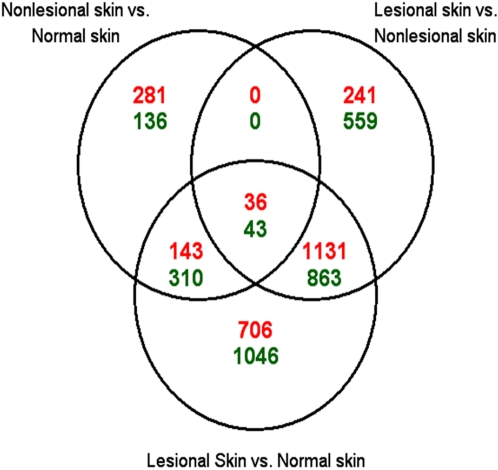
Probe sets found to be altered using microarray analysis. Venn diagram illustrates the number of probe sets that show altered expression at the mRNA level in all three combinations of lesional skin, nonlesional skin, and skin from healthy donors. Values shaded in red indicate the number of probe sets significantly up-regulated; values shaded in green indicate the number of probe sets significantly down-regulated. The intersecting regions represent probe sets that are common to the specific comparisons.

Additionally, we observed that 460 probe sets were up-regulated and 489 probe sets were down-regulated in nonlesional skin compared with skin from healthy donors. The genes differentially regulated in nonlesional skin are mostly distinct from those altered in lesional skin. Figure 1 shows a Venn diagram of the probe sets altered in lesional skin and nonlesional skin. Only 36 of the 1408 up-regulated probe sets in lesional skin were also up-regulated in nonlesional skin. Similarly, only 43 of the 1465 probe sets down-regulated in lesional skin were also down-regulated in nonlesional skin. Taken together, these data suggest that the molecular events and biological changes from nonlesional skin to lesional skin are mainly distinct from the differences between normal and nonlesional skin.

Of the genes up-regulated in lesional skin, CD4, CD69, IL-7R, and IL-2Rγ are T cell markers; CXCL9, CXCL10, CXCL11, CCL18, CCL19, and CCR7 are involved in T cell trafficking and recruitment; LAMP3, CD83, CD163, CD53, and CD24 are dendritic cell surface markers; IL-1β, IRAK2, IL-1F5, IL-1F6, and IL-1F9 are IL-1–inducible genes; IL-8 is a neutrophil-chemotactic factor whose production can be induced by IL-1, TNF-α and OSM. The strong overexpression of S100A family members and DEFB4 in lesional skin could result from both neutrophil infiltration and keratinocyte activation. Overall, these observations agree with the well-known presence of T cells, dendritic cells, and neutrophils in lesional skin of psoriatic patients. It is also worth noting that IL-8 mRNA is highly up-regulated in lesional skin, whereas it is slightly down-regulated in nonlesional skin compared with skin from healthy donors (Table S1). This observation suggests an increase of neutrophil activity in lesional skin of psoriatic patients.

Many of the genes downregulated in nonlesional skin compared to normal skin are transcription factors such as JUN, JUNB, FOSB, ATF3, NR4A2, PER1, EGR1, MAFF (Table S3). This finding suggests that nonlesional skin, although overtly normal, has readily identifiable alterations at the transcript level. In contrast, the most downregulated genes in lesional skin compared to nonlesional skin include genes that encode structural, cell adhesion and tight junction proteins such as CLDN8, KRT1B, CNTNAP3B, PCDH21, PAPLN; immune response genes such as IL1F7, CCL27, F3; and genes involved in signaling pathways such as WIF1, ADRB2, TIMP3 (Table S2).

**Figure 2:**
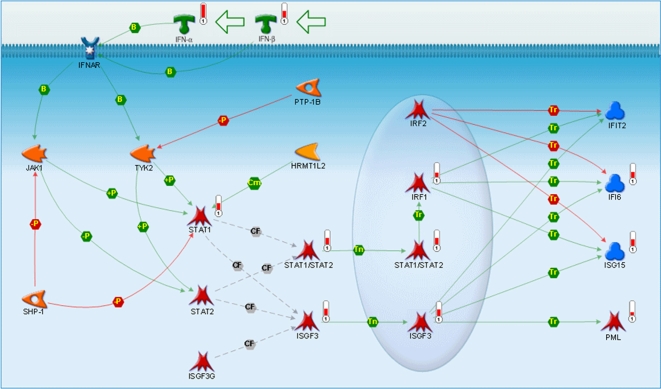
Type I IFN signaling pathway activated in lesional skin of psoriatic patients. Pathway image was generated with GeneGo's MetaCore integrated software suite. Individual symbols within the image represent well–characterized proteins or protein complexes. Arrows linking the proteins represent the stimulatory, inhibitory, or interactive effect of the protein on the target protein. Thermometers adjacent to the individual symbols represent relative expression levels (red indicates overexpression; blue indicates underexpression) of transcripts that encode the protein (or protein complex) within the particular pathway.

To identify the signaling pathways altered in psoriasis, we performed an analysis using the GeneGo MetaCore pathway and network approach (see Materials and Methods). Overall, 22 signaling pathways were activated and 37 signaling pathways were suppressed (*P*<0.05) in lesional skin compared with nonlesional skin. All the putative signaling pathways activated were cytokine- and chemokine-mediated signaling pathways or were involved in immune responses. For example, in agreement with the literature [10], [12], IFN-γ, TNF-α, and OSM signaling pathways were activated in lesional skin of psoriatic patients. Of all the signaling pathways altered between lesional and nonlesional skin, the IFN-α/β signaling pathway was the most dominant and significant pathway (*P* = 6.6×10^−26^; Figure 2). Components of the pathway, such as IFNAR1, IFNAR2, STAT1, IRF1, MPL, ISG15, and IFI6, were all significantly overexpressed in lesional skin compared with nonlesional skin of psoriatic patients. WNT, PTEN, PDGF, ESR1 and several cell adhesion pathways are among the most significantly suppressed pathways in lesional skin compared to nonlesional skin of psoriatic patients.

### Type I IFN–inducible genes are overexpressed in lesional skin of psoriatic patients

To determine the prevalence and magnitude of the overexpression of type I IFN–inducible genes in psoriasis, we first carried out ex vivo stimulation of healthy donor whole blood and in vitro stimulation of normal human keratinocytes with different members of the type I IFN family (see Materials and Methods and Supplemental Material) to identify type I IFN–inducible genes. We observed 689 probe sets (about 1.3% of all probe sets on the Affymetrix human genome U133 plus v2.0 array) that were uniformly up-regulated in whole blood following stimulation for 4 hours by 1 of the 9 IFN-α subtypes or by IFN-β. A total of 336 probe sets were down-regulated by IFN-α or IFN-β in the same experiment. In keratinocytes, many of these genes (i.e., IFIT1, IFIT2, IFIT3, RSAD2, ISG15, MX1, DDX58, IFI44, IFI44L) were also up-regulated following in vitro stimulation by IFN-α2a, an IFN-α subtype, or by leukocyte IFN (a mixture of type I IFNs). The top 50 probes upregulated by leukocyte IFN are listed in Table S4. The probes upregulated by IFN-α2a are similar to those by leukocyte IFN (data not shown).

From the list of probe sets that were identified to be type I IFN–inducible from the ex vivo stimulation studies, we identified 164 of 1408 probe sets (11.7%) that were up-regulated in lesional skin compared with nonlesional skin as being type I IFN-inducible. Using Fisher exact test (1-tailed *t* test), the observed overexpression of type I IFN–inducible genes in lesional skin of psoriatic patients was found to be statistically significant (*P*<0.0001). We also observed that 19 of the top 100 (Table S1) most up-regulated probe sets in lesional skin were type I IFN–inducible genes, indicating an enrichment of type I IFN–inducible genes among the most overexpressed genes in lesional skin of psoriatic patients. Table 1 lists the top 50 most up-regulated type I IFN–inducible genes (expressed sequence tags [ESTs] and hypothetical proteins are excluded from the list) in lesional skin of psoriatic patients.

**Table 1:** Fold Changes (fc; log_2_ transformed) and q Values (Calculated by FDR) for the Top 50 Most Up-regulated Type I IFN-Inducible Genes in Psoriatic Lesional Skin Versus Nonlesional Skin.

				Lesional vs Nonlesional		Nonlesional vs Normal	
Probe ID	Unigene ID	Gene Title	Gene Symbol	log2.fc	q value	log2.fc	*q* Value
219403_s_at	Hs.44227	heparanase	HPSE	4.598	4.46E-22	0.226	0.2359
204972_at	Hs.414332	2′-5′-oligoadenylate synthetase 2, 69/71 kDa	OAS2	4.098	8.57E-14	0.096	0.289
205660_at	Hs.118633	2′-5′-oligoadenylate synthetase-like	OASL	4.03	1.34E-12	0.029	0.2034
227609_at	Hs.546467	epithelial stromal interaction 1 (breast)	EPSTI1	4.002	1.14E-14	−0.254	0.108
227458_at	—	—	—	3.859	9.31E-14	−0.591	0.0545
219352_at	Hs.529317	hect domain and RLD 6	HERC6	3.842	9.49E-16	−0.46	0.0481
216834_at	Hs.75256	regulator of G-protein signalling 1	RGS1	3.809	2.47E-17	−5.269	0
204533_at	Hs.632586	chemokine (C-X-C motif) ligand 10	CXCL10	3.697	2.97E-12	0.338	0.1302
226702_at	Hs.7155	hypothetical protein LOC129607	LOC129607	3.572	2.37E-16	−0.156	0.265
242625_at	Hs.17518	radical S-adenosyl methionine domain containing 2	RSAD2	3.403	1.65E-12	−0.07	0.3131
213797_at	Hs.17518	radical S-adenosyl methionine domain containing 2	RSAD2	3.243	3.36E-10	0.004	0.3621
202086_at	Hs.517307	myxovirus (influenza virus) resistance 1	MX1	3.235	5.28E-14	0.05	0.3345
205552_s_at	Hs.524760	2′,5′-oligoadenylate synthetase 1, 40/46 kDa	OAS1	3.222	2.41E-14	0.328	0.1367
210797_s_at	Hs.118633	2′-5′-oligoadenylate synthetase-like	OASL	3.216	1.63E-09	0.005	0.3494
204439_at	Hs.389724	interferon-induced protein 44-like	IFI44L	3.205	4.73E-13	0.12	0.3007
202411_at	Hs.532634	interferon, alpha-inducible protein 27	IFI27	3.165	4.81E-12	−0.154	0.2688
202869_at	Hs.524760	2′,5′-oligoadenylate synthetase 1, 40/46 kDa	OAS1	3.15	2.47E-14	0.248	0.214
205483_s_at	Hs.458485	ISG15 ubiquitin-like modifier	ISG15	3.088	4.73E-13	−0.273	0.1101
209969_s_at	Hs.565365	signal transducer and activator of transcription 1, 91 kDa	STAT1	2.993	7.95E-17	0.199	0.2007
228531_at	Hs.65641	sterile alpha motif domain containing 9	SAMD9	2.846	5.42E-14	−0.033	0.3536
204415_at	Hs.523847	interferon, alpha-inducible protein 6	IFI6	2.769	7.23E-09	−0.045	0.2907
214453_s_at	Hs.82316	interferon-induced protein 44	IFI44	2.679	1.94E-12	0.086	0.3262
222838_at	Hs.517265	SLAM family member 7	SLAMF7	2.659	1.60E-16	−0.046	0.3122
219684_at	Hs.43388	receptor transporter protein 4	RTP4	2.649	3.73E-11	0.497	0.0491
203127_s_at	Hs.435661	serine palmitoyltransferase, long chain base subunit 2	SPTLC2	2.628	1.04E-20	−1.016	0.0002
205569_at	Hs.518448	lysosomal-associated membrane protein 3	LAMP3	2.569	2.64E-09	0.293	0.2287
219691_at	Hs.65641	sterile alpha motif domain containing 9	SAMD9	2.559	1.30E-13	0.011	0.3735
223220_s_at	Hs.518200	poly (ADP-ribose) polymerase family, member 9	PARP9	2.553	1.08E-15	0.069	0.3142
AFFX-HUMISGF3A/M97935_MA_at	Hs.565365	signal transducer and activator of transcription 1, 91 kDa	STAT1	2.525	1.64E-10	0.706	0.0334
212268_at	Hs.381167	serpin peptidase inhibitor, clade B (ovalbumin), member 1	SERPINB1	2.51	3.02E-15	−0.605	0.0775
216202_s_at	Hs.435661	serine palmitoyltransferase, long chain base subunit 2	SPTLC2	2.507	1.17E-13	−0.682	0.0169
229450_at	—	—	—	2.492	1.50E-14	0.224	0.2067
208436_s_at	Hs.166120	interferon regulatory factor 7	IRF7	2.448	6.90E-15	−0.578	0.0161
AFFX-HUMISGF3A/M97935_5_at	Hs.565365	signal transducer and activator of transcription 1, 91 kDa	STAT1	2.444	3.03E-10	0.516	0.0585
204747_at	Hs.47338	interferon-induced protein with tetratricopeptide repeats 3	IFIT3	2.424	2.15E-14	0.365	0.0722
229390_at	Hs.381220	hypothetical protein LOC441168	RP1-93H18.5	2.4	2.59E-12	−0.369	0.1143
218400_at	Hs.528634	2′-5′-oligoadenylate synthetase 3, 100 kDa	OAS3	2.397	3.83E-14	0.179	0.1163
235276_at	—	—	—	2.386	3.61E-15	0.057	0.3277
203153_at	Hs.20315	interferon-induced protein with tetratricopeptide repeats 1	IFIT1	2.351	1.17E-10	0.054	0.3445
210873_x_at	Hs.348983	apolipoprotein B mRNA editing enzyme, catalytic polypeptide-like 3A	APOBEC3A	2.348	1.35E-07	−0.048	0.3012
204698_at	Hs.459265	interferon stimulated exonuclease gene 20 kDa	ISG20	2.337	1.50E-12	−0.644	0.0505
232666_at	Hs.528634	2′-5′-oligoadenylate synthetase 3, 100 kDa	OAS3	2.236	4.50E-10	0.077	0.0482
222881_at	Hs.44227	heparanase	HPSE	2.23	3.47E-15	0.221	0.1713
205241_at	Hs.567405	SCO cytochrome oxidase deficient homolog 2 (yeast)	SCO2	2.208	1.90E-17	−0.285	0.0852
AFFX-HUMISGF3A/M97935_MB_at	Hs.565365	signal transducer and activator of transcription 1, 91 kDa	STAT1	2.205	5.29E-10	0.397	0.1022
206553_at	Hs.414332	2′-5′-oligoadenylate synthetase 2, 69/71 kDa	OAS2	2.183	1.34E-09	0.043	0.1476
207387_s_at	Hs.1466	glycerol kinase	GK	2.16	9.38E-14	0.014	0.3749
219716_at	Hs.257352	apolipoprotein L, 6	APOL6	2.123	3.03E-11	−0.126	0.1925
202270_at	Hs.62661	guanylate binding protein 1, interferon-inducible, 67 kDa	GBP1	2.113	4.67E-14	−0.053	0.3137
AFFX-HUMISGF3A/M97935_3_at	Hs.565365	signal transducer and activator of transcription 1, 91 kDa	STAT1	2.11	3.93E-17	−0.148	0.1286

Also listed are the log_2_ transformed fold changes and q values for these genes when comparing nonlesional skin with 21 healthy skin controls. Data were generated from 26 paired nonlesional and lesional skin (3 nonlesional skin biopsies were dropped from analysis due to technical issues, see Materials and Methods) from 24 psoriatic patients and 5 lesional skin biopsies from 3 psoriatic patients using SAM and FDR in R (see Materials and Methods). The GC-RMA normalized raw microarray data is available at: http://www.medimmune.com/translationalscience/data/psoriasis2007.

**Figure 3:**
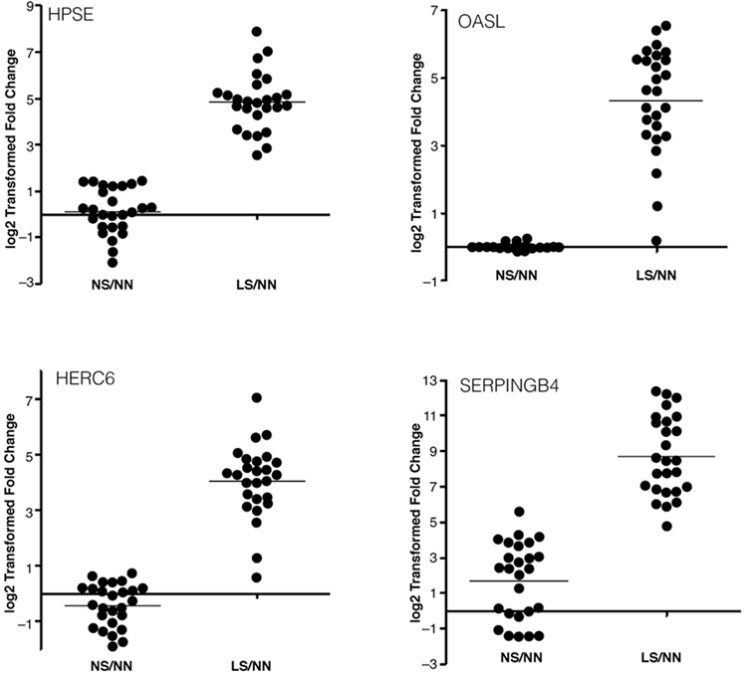
Relative expression of selected genes. Shown are three type I IFN–inducible genes (HPSE, OASL, and HERC6) and 1 gene that is not type I IFN–inducible (SERPINB4) in lesional skin (LS) compared with nonlesional skin (NS) in psoriatic patients, and in NS compared with skin from healthy donors (NN) based on microarray data. The fold changes of these genes in LS are compared with their paired NS, and NS is compared with the average of 21 NN controls. The horizontal bars represent the average fold change. The *P* values for the relative expression of HPSE, OASL, HERC6, and SERPINB4 between NS and NN, and between LS and NS are (listed in pairs): 0.468, <0.00001; 0.376, <0.00001; 0.03, <0.00001; 0.0002, <0.00001, respectively.

Next, we asked whether type I IFN–inducible genes are also significantly overexpressed in nonlesional skin of psoriatic patients compared with skin from normal donors. Of all the probe sets that are overexpressed in nonlesional skin of psoriatic patients compared with skin from healthy donors, only 1% (5 probe sets) are type I IFN–inducible. The overexpression of type I IFN–inducible genes was not significant (Fisher exact test [2-tailed *t* test]; *P* = 0.581) in nonlesional skin of psoriatic patients. From Table 1, we observe that for those genes up-regulated in lesional skin compared with nonlesional skin in psoriatic patients, nearly all are expressed at similar levels in nonlesional skin compared with skin from healthy donors (several genes [eg, RGS1 and SPTLC2] are down-regulated in nonlesional skin compared with skin from healthy donors). This observation was also confirmed by pathway analysis in which the IFN-α/β signaling pathway was not significantly altered in nonlesional skin compared with skin from healthy donors (*P* value close to 1). Figure 3 shows the relative expression of 3 type I IFN–inducible genes (HPSE, OASL, and HERC6) and 1 gene that is not IFN–inducible (SERPINB4) in lesional skin compared with nonlesional skin, and in nonlesional skin compared with skin from healthy donors. The expression of these genes in lesional skin compared with nonlesional skin is statistically significant, and on average, 12- and 250-fold higher,, whereas the mRNA levels of these genes remain unaltered in nonlesional skin compared with skin from healthy donors.

**Figure 4:**
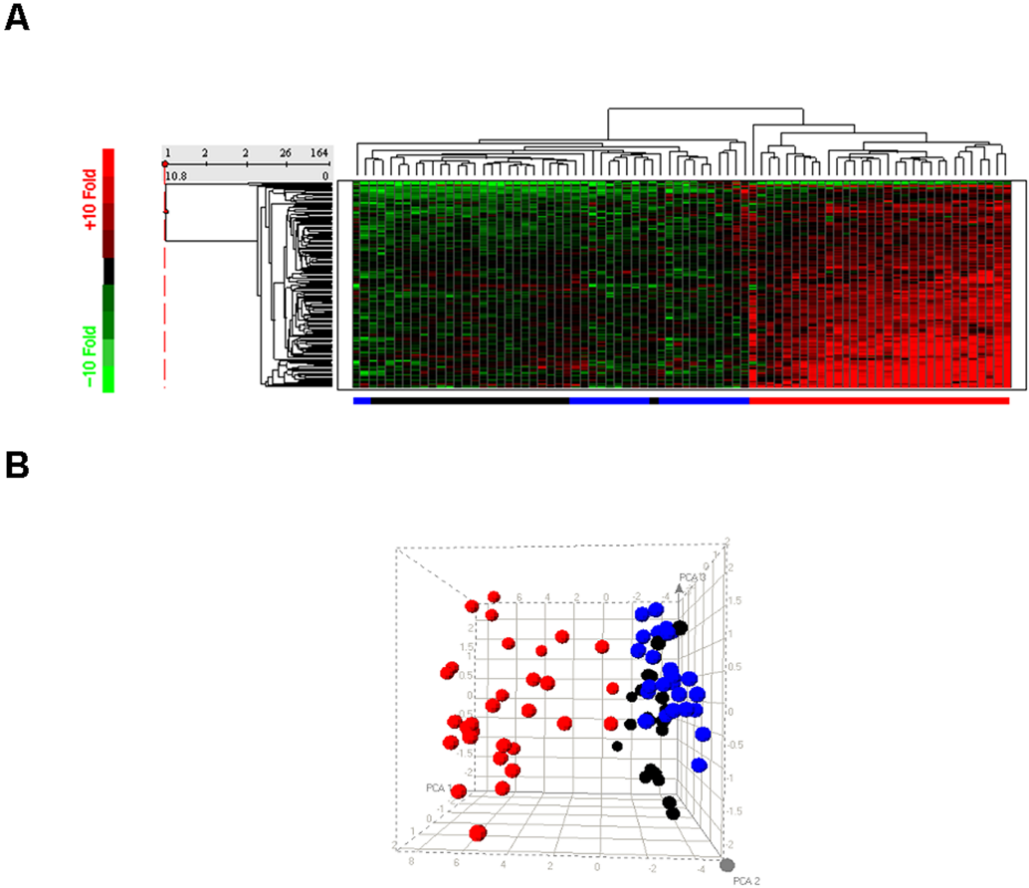
Clustering of up-regulated genes. A, Hierarchical clustering of all psoriasis samples profiled (blue: 21 normal, black: 23 paired nonlesional, and pink: 26 lesional skin from psoriatic patients; and red: 5 unpaired lesional skin from 3 psoriatic patients) using the 164 up-regulated type I IFN–inducible probe sets in lesional skin compared with those in mostly paired nonlesional skin. Each row corresponds to a single probe set; each column corresponds to a single sample. Branch lengths indicate the degree of similarity with joined samples, with a longer branch indicating a less similarity between joined samples. Red represents up-regulation vs control (ie, average expression of 21 normal samples), and green represents down-regulation vs control. B, 3-dimensional PCA plot of all psoriasis samples profiled using the 164 up-regulated type I IFN–inducible probe sets in lesional skin compared with those in mostly paired nonlesional skin. Each point represents 1 sample (blue: skin from healthy donors, black: nonlesional skin, red: lesional skin).

Figure 4A shows a heat map of unsupervised hierarchical clustering of the 164 up-regulated type I IFN–inducible probe sets in all lesional and nonlesional skin from psoriatic patients and skin from healthy donors. These samples segregate into 2 large clusters: one composed of all lesional skin biopsies and the other composed of all normal and nonlesional skin samples. This observation demonstrates that the magnitude of the overexpression of type I IFN–inducible genes in lesional skin is distinct from that in nonlesional skin or skin from healthy donors.

Figure 4B shows the principal components analysis (PCA) plot of the same samples shown in Figure 4A using the same 164 type I IFN–inducible probe sets. PCA is a mathematical calculation that reduces the dimensionality of the data set so that each individual sample can be viewed in a 2- or 3-dimensional plot. Skin biopsies from healthy donors and nonlesional skin biopsies are mostly clustered together, which suggests that they have comparable level of expression of type I IFN–inducible genes. Furthermore, the analysis shows that the majority of lesional skin biopsies clearly separate from normal and nonlesional skin biopsies. This indicates that most of these patients had overexpression of the type I IFN–inducible genes, and that was distinct from those in the other 2 groups. Three lesional skin biopsies are close to the nonlesional skin and skin from healthy donors group in the PCA plot. These 3 samples were from the 3 psoriatic patients that had weaker overexpression of type I IFN–inducible genes compared with the rest of the psoriatic patients in the study.

We then asked whether the observed overexpression of the type I IFN–inducible genes in psoriatic patients from microarray studies could be confirmed by a second platform. We used a BioMark™ 48.48 dynamic array, a high throughput TaqMan quantitative real-time polymerase chain reaction (QRT-PCR)–based technology from Fluidigm, to analyze the profiled samples. Lesional and nonlesional skin biopsy pairs from 18 psoriatic patients, along with pooled biopsies from healthy donors, were assayed for the relative expression of mRNA of 40 genes (29 genes were type I IFN–inducible; the other 11 genes were among the most up-regulated in lesional skin of psoriatic patients). These genes were selected based on their prevalence and magnitude of overexpression in lesional skin biopsies. The overexpression of all genes in lesional skin was confirmed by TaqMan QRT-PCR. In the majority of the genes, good correlation between microarray and TaqMan assays was observed [13]. Overall, these observations further substantiate that type I IFN–inducible genes are significantly overexpressed in lesional skin of psoriatic patients but not in nonlesional skin of patients or skin from normal donors.

### Type I IFNs, IFNAR1, and IFNAR2 mRNA are up-regulated in lesional skin as compared with nonlesional skin and skin from healthy donors

Given that we observed significant overexpression of type I IFN–inducible genes in lesional skin of psoriatic patients, we wanted to characterize the type I IFNs that may be responsible. As reported previously, we were not able to detect IFN-α proteins in skin from healthy donors or stable psoriatic skin [14]. Schmid et al [15] were able to use in situ hybridization to weakly detect IFN-α mRNA throughout the hyperkeratotic epidermis. However, they could not detect IFN-β, most likely because of the sensitivity limitation in their measurements.

**Figure 5:**
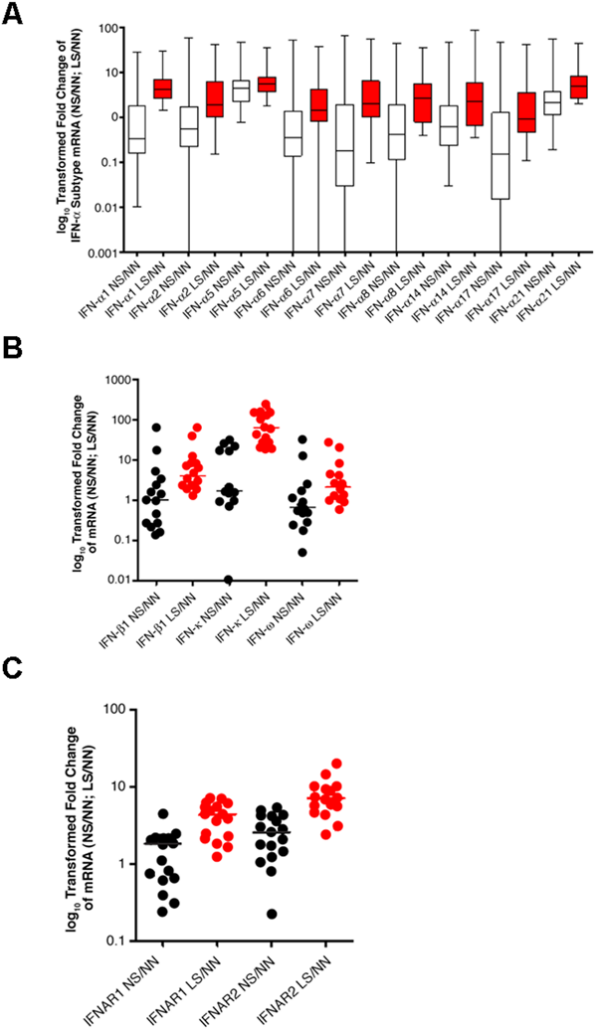
Relative expression and median fold changes of select mRNAs. Selected mRNAs include (A) Type I IFN-α subtypes (box plot), (B) other Type-I IFNs, and (C) IFN-α receptors in lesional skin (LS) or nonlesional skin (NS) compared with skin from healthy donors (NN). Horizontal bars represent median fold change. Black points: relative fold change in NS vs NN (NS/NN); Red points: relative fold change in LS vs NN (LS/NN). The *P* values for the overexpression of these genes in NS or LS vs NN (listed in pairs) are as follows: IFN-α1, 0.303, <0.001; IFN-α2, 0.389, 0.072; IFN-α5, <0.001, 0.002; IFN-α6, 0.664, 0.093; IFN-α7, 0.586, 0.077; IFN-α8, 0.430, 0.049; IFN-α14, 0.224, 0.049; IFN-α17, 0.552, 0.0203; IFN-α21, 0.113, 0.003; IFN-β, 0.255, 0.022; IFN-κ, 0.03, <0.001; IFN-ω, 0.516, 0.049; IFNAR1, 0.192, <0.001; IFNAR2, <0.001, <0.001, respectively.

We used the TaqMan Low Density Array (TLDA) technology (a TaqMan QRT-PCR–based assay) from Applied Biosystems to measure the mRNA level of type I IFN family members in paired lesional skin and nonlesional skin from 18 different psoriatic patients, together with RNA from 2 of the healthy donor skin biopsies. Genes that represent 9 IFN-α subtypes (1, 2, 5, 6, 7, 8, 14, 17 and 21; the primers for 4 other IFN-α subtypes were not available), IFN-β, -κ, -ω, IFNAR1, and IFNAR2 were printed on the array. The relative overexpression of mRNA of 9 IFN-α subtypes in lesional skin compared with either nonlesional skin or skin from healthy donors is shown in Figure 5A. With the exception of IFN-α5 (*P*<0.001), none of the mRNA levels of the IFN-α subtypes were significantly altered in nonlesional skin compared with skin from healthy donors. However, all of these IFN-α subtypes were up-regulated at the mRNA level in lesional skin compared with that in skin from healthy donors. The overexpression of IFN-α1, IFN-α5, IFN-α8, IFN-α14, IFN-α17, IFN-α21 was statistically significant (*P*<0.05). The overexpression of the mRNAs of other members of the type I IFN family, (IFN-β, -κ, and –ω) in lesional skin compared with nonlesional skin or skin from healthy donors is shown in Figure 5B. The alterations of IFN-β and IFN-ω mRNAs in nonlesional skin were not significant. However, these mRNAs were significantly up-regulated in lesional skin compared with skin from healthy donors (*P* = 0.022 and *P* = 0.049, respectively). IFN-κ mRNA was up-regulated by approximately 1.6-fold (median fold change) in nonlesional skin (*P* = 0.03), and was sharply up-regulated by 62.6-fold (median fold change) in lesional skin compared with skin from healthy donors (*P*<0.001). Furthermore, the receptors for type I IFNs, IFNAR1 and IFNAR2, were also significantly overexpressed in lesional skin of psoriatic patients at the transcript level (*P*<0.001; Figure 5C). Although IFNAR2 up-regulation was significant in nonlesional skin, up-regulation of IFNAR1 was not (Figure 5C). Collectively, these data provided evidence that mRNA levels for most type I IFN family members were comparable between nonlesional skin and skin from healthy donors (with the exception of IFN-α5 and IFN-κ) and were significantly increased in lesional skin of psoriatic patients.

### Co-overexpression of type I, type II IFN, and TNF-α and their gene signatures in lesional skin of psoriatic patients

T cells contribute to the development of diseased skin in psoriasis and to the epidermal hyperproliferation in genetically predisposed individuals through the secretion of Th1 cytokines. Since biologics that target T cells and TNF-α show clinical benefit in psoriasis, we addressed the involvement of IFN-γ and TNF-α in the pathogenesis of the disease. As mentioned earlier, we observed the activation of both the IFN-γ and TNF-α signaling pathways in lesional skin of psoriatic patients.

**Figure 6:**
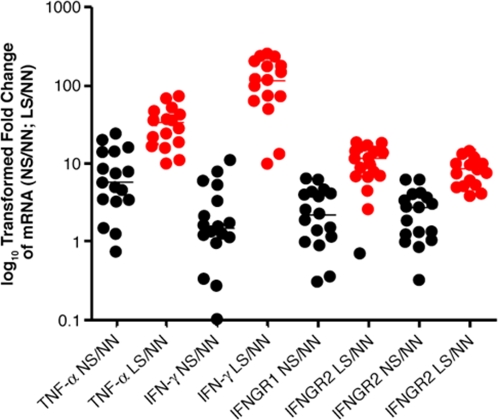
Changes in expression of TNF-α, IFN-γ, and IFN-γ receptors. Relative expression of mRNA and median fold changes of in lesional skin (LS), or nonlesional skin (NS) compared with skin from healthy donors (NN) are shown. The averages of the relative mRNA levels of these cytokine and their receptors in NN from 2 donors were scaled to 1 based on TaqMan QRT-PCR assays. The horizontal bars represent the median fold change. Black points: the relative fold change of mRNA in NS compared with NN; Red points: the relative fold change of mRNA in LS compared with NN. The *P* values for the overexpression of these individual genes in NS or LS compared with NN (listed in pairs) are as follows: IFN-γ, 0.02, <0.001; IFNGR1, <0.001, <0.001; IFNGR2, <0.001, <0.001; TNF-α, <0.001, <0.001, respectively.

We used TLDA to measure the mRNA levels of IFN-γ, IFNGR1, IFNGR2 and TNF-α in lesional and nonlesional skin from psoriatic patients and compared them with those from healthy donors. Unlike what was found with type I IFNs, the mRNA levels of IFN-γ, IFNGR1, IFNGR2, and TNF-α were significantly overexpressed in nonlesional skin compared with skin from healthy donors (Figure 6; *P* = 0.02, *P*<0.001, *P*<0.001 and *P*<0.001 respectively). These genes were further up-regulated in lesional skin compared with either nonlesional skin (*P* = 0.04, *P* = 0.01, *P* = 0.001 and *P* = 0.007 respectively) or skin from healthy donors (*P*<0.001 for all; Figure 6). These results show that expression of mRNA for IFN-γ and TNF-α is different from that of type I IFN family members.

**Figure 7:**
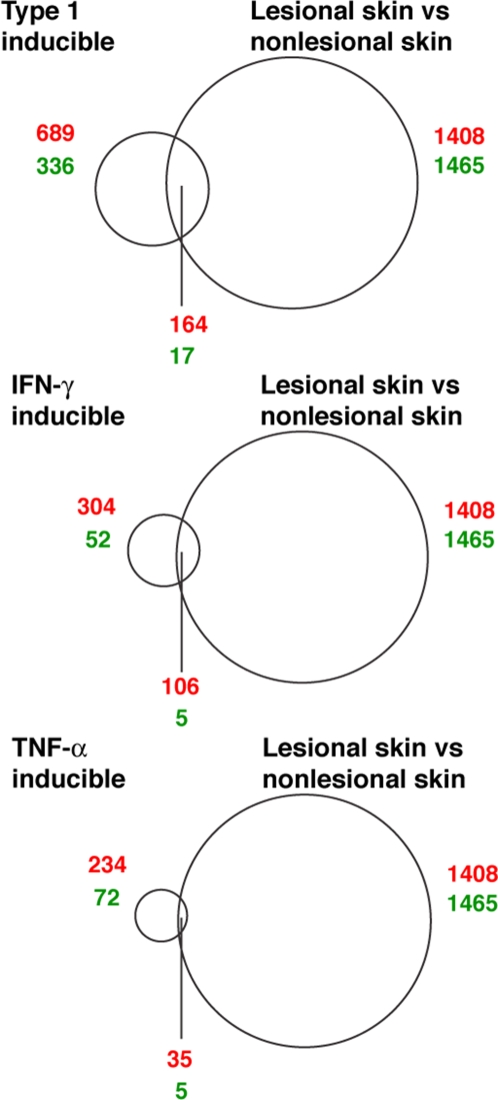
Probe sets common to lesions and ex vivo stimulation. Venn diagrams illustrating both the number of probe sets that are altered by type I IFN, IFN-γ, and TNF-α in ex vivo stimulation of whole blood from healthy donors (left circles) and probe sets that are altered in lesional skin compared with nonlesional skin (right circles). Red numbers: probe sets that show increased expression with cytokine treatment or compared with nonlesional skin baseline. Green numbers: probe sets that show decreased expression with cytokine treatment or compared with nonlesional skin baseline. The intersecting regions represent the probe sets that are common to both comparisons.

The ex vivo stimulation of healthy donor whole blood and in vitro stimulation of keratinocytes with IFN-γ and TNF-α was used to identify genes induced by these cytokines. A total of 304 and 234 probe sets were up-regulated by IFN-γ and TNF-α, respectively, by at least 2-fold in the whole blood of all 4 donors challenged for 4 hours. Of the 1408 probe sets that were up-regulated in lesional skin compared with nonlesional skin of psoriatic patients, 106 and 35 of them were IFN-γ and TNF-α–inducible, respectively (Figure 7). Fisher exact test (1-tailed *t* test) indicated that overexpression of IFN-γ and TNF-α–inducible genes in lesional skin was significant (*P*<0.0001).

**Figure 8:**
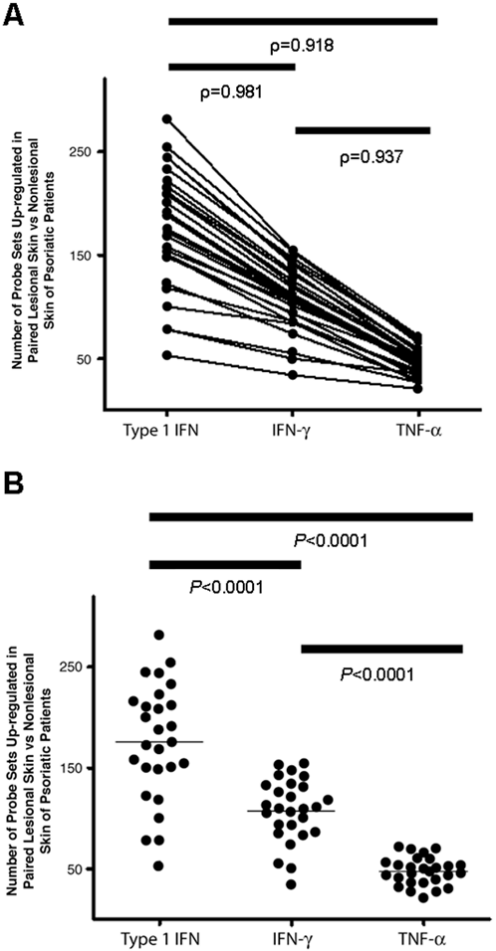
Co-overexpression of type I IFN, IFN-γ and TNF-α–inducible genes. Paired lesional and nonlesional skin of psoriatic patients was analyzed with Affymetrix Genechip®. The type I IFN, IFN-γ and TNF-α–inducible genes were selected based on healthy donor whole blood ex vivo stimulation with these cytokines. A probe set with fold change of ≥2 in lesional skin compared with its paired nonlesional skin is considered overexpressed in the specific lesional skin. A, The number of up-regulated type I IFN–, IFN-γ– and TNF-α–inducible genes in lesional skin compared with paired nonlesional skin shows a strong correlation (ρ = Pearson's correlation coefficient) from pairwise comparisons. B, The numbers of type I IFN–, IFN-γ– and TNF-α–inducible genes in lesional skin compared with nonlesional skin are significantly different as evident from pairwise comparisons. The horizontal bars represent the median value for each group.

Type I IFN, IFN-γ, and TNF-α–inducible probe sets identified in the ex vivo study allowed us to identify the type I IFN, IFN-γ, and TNF-α–inducible genes in each lesional skin biopsy compared with paired nonlesional biopsies that were up-regulated by at least 2-fold. Figure 8A shows the number of type I IFN, IFN-γ, and TNF-α–inducible genes up-regulated in each of the 26 paired lesional and nonlesional skin biopsies. The numbers of IFN-γ and TNF-α–inducible genes in a given lesional skin biopsy positively correlated with the number of type I IFN–inducible genes in the same sample. This observation was confirmed by the strong correlation in the coactivation of these 3 sets of genes with correlation coefficients of at least 0.918 in all 3 pairwise comparisons (Figure 8A) and by significant *P* values when comparing the up-regulation of any 2 of the 3 sets of genes in lesional skin compared with nonlesional skin (*P*<0.001 for all 3 comparisons; Figure 8B).

A similar analysis was carried out for genes that were down-regulated in lesional skin compared with the nonlesional skin of psoriatic patients (Figure 7). Of the 1465 probe sets down-regulated in lesional skin, only 17, 5, and 5 of them were type I IFN, IFN-γ, and TNF-α inducible.

Somewhat surprisingly we found that although mRNAs of IFN-γ and TNF-α were found to be up-regulated in nonlesional skin of psoriatic patients when compared with those from healthy donors, we did not find that IFN-γ or TNF-α–inducible genes were significantly overexpressed in nonlesional skin (data not shown). The up-regulation of mRNAs of type I IFNs, IFN-γ, and TNF-α, together with the corresponding up-regulation of their respective gene signatures in psoriatic lesional skin, suggests the presence of type I IFNs, IFN-γ, and TNF-α protein and active signaling in skin lesions. In contrast, the elevated expression of mRNAs of IFN-γ and TNF-α in nonlesional skin does not result in corresponding up-regulation of IFN-γ or TNF-α–inducible genes. This suggests that IFN-γ and TNF-α proteins are not made in nonlesional skin or that other signaling molecules might have inhibitory effects on the IFN-γ and TNF-α pathways in nonlesional skin of psoriatic patients.

### Immunohistochemical analysis of psoriatic, nonlesional skin, and skin from healthy donors

**Figure 9:**
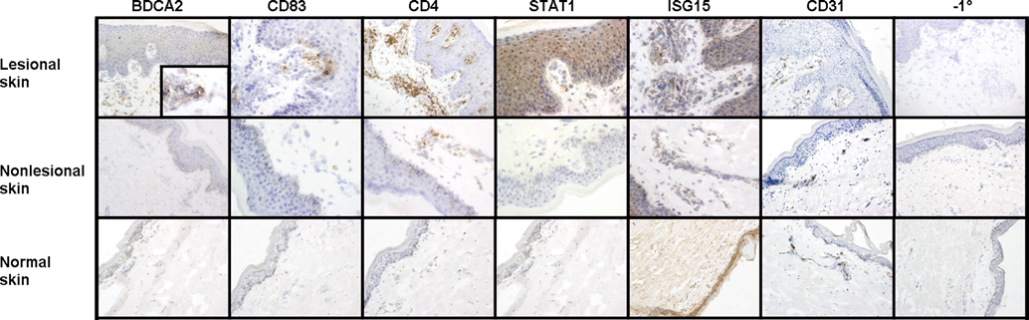
Immunohistochemical analyses of biopsies from psoriatic lesional and nonlesional skin and skin from normal donors. BDCA2 is a specific marker for pDCs, which are present at greater numbers in lesional skin (inset is 100×) compared with nonlesional skin, and not at all in skin from healthy donors. CD83 is a marker for mDCs; CD4 is present on T cells and dendritic cells. STAT1 protein staining was observed in the epidermis of lesional skin (both nuclear and cytoplasmic) and dermal mononuclear inflammatory cells, but not in nonlesional or skin from healthy donors. ISG15 protein increase was observed in psoriatic lesional skin and to a lesser extent in nonlesional skin but was not detected in the dermis of healthy donors. A positive tissue control (CD31) and an assay control (−1°) was included that eliminated the primary antibody from the immunohistochemical assay.

To determine whether the highly overexpressed type I IFN–inducible genes in psoriatic skin gave rise to similar changes in the expression of the proteins, immunohistochemical analyses were carried out to assess the presence of 2 type I IFN–inducible proteins, STAT1 and ISG15, in skin from the same patients and the same biopsy samples that underwent whole genome array analysis as described above. Furthermore, immunohistochemical characterization of the cellular infiltrates (pDCs, mDCs, and CD4^+^ cells) was conducted to compare the number of IFN–producing cell types and inflammatory cells in the biopsies of lesional versus nonlesional and skin from healthy donors. In all patients with evaluable biopsy pairs, lesional skin contained increased numbers of CD4^+^ cells, as well as significant up-regulation of STAT1 and ISG15 protein in the epidermis and dermis when compared with nonlesional biopsies. Of the 16 pairs of lesional and nonlesional skin biopsies, 6 pairs showed increased numbers of pDCs, and 9 pairs showed increased numbers of mDCs in lesional skin compared with nonlesional skin. Although nonlesional skin appeared grossly normal, there were often microscopic morphological changes that included low numbers of infiltrating mononuclear cells, including pDCs, mDCs, and CD4^+^ cells. In contrast, staining for pDCs, mDCs, STAT1, and dermal ISG15 gave no appreciable signal in skin from healthy donors; however, CD4^+^ cells were rarely observed in the sections of normal skin examined. CD31 was used as a positive tissue control, to demonstrate that expressed epitopes in the skin cryosections could be detected by immunohistochemical staining (Figure 9).

## Discussion

Type I IFNs are produced in response to viral and bacterial infections and play a key role in host defense mechanisms. They stimulate the maturation of pDCs and the generation and function of natural killer (NK), T, and B cells [16], [17]. The type I IFN family is composed of several family members (13 IFN-α subtypes, IFN-β, -ω, -κ, -σ, -δ, -λ); IFN-α and IFN-β are the most abundant. IFN-γ, a type II IFN, is mainly produced by activated T cells, dendritic cells, and NK cells and is involved in adaptive immune responses.

IFNs have long been associated with the pathogenesis of psoriasis. IFN pathways are active in psoriatic lesions [18], [19]. Treatment of non-psoriatic disease with IFN can induce or exacerbate psoriasis [20]–[23], as can treatment with imiquimod, a toll-like receptor agonist that induces production of type I IFNs from pDCs [24]. Constitutive IFN-α/β signaling in mice lacking IFN regulatory factor-2 induces a psoriasis-like inflammatory skin disease [25]. A direct functional role of IFN-α/β in psoriasis initiation was recently demonstrated in a xenograft murine model of human psoriasis, where nonlesional skin from psoriatic patients was transplanted onto immunocompromised mice [26]. Disruption of IFN-α signaling or the inhibition of the ability of pDCs to produce IFN-α prevented the T cell–dependent development of psoriasis,[26]. Thus, pDC-derived IFN-α is both necessary and sufficient for disease manifestation in this model; however, confirmatory human data is lacking.

Microarray analysis of disease tissues has widely been used to identify etiopathogenic factors of disease activity, and previous studies have used microarray analyses to profile lesional skin and nonlesional skin from psoriatic patients [7], [27]. However, neither of these studies was performed using a whole genome array platform. Furthermore, the first study lacked skin from healthy donors as a control and was carried out on a limited number of patient samples. The second study focused mainly on T cell and dendritic cell activation in lesional skin of psoriatic patients.

The data described here represent the first large-scale study on human samples that demonstrates the prevalence of type I IFNs and type I IFN–inducible genes in psoriatic skin. We show that (1) mRNAs of type I IFN family members are up-regulated in lesional skin but not in nonlesional skin (except IFN-α5 and IFN-κ); (2) IFN-α/β signaling pathway is the most significantly activated pathway in lesional skin compared with both nonlesional skin and skin from healthy donors; (3) there is a prevalent and robust overexpression of a large panel of type I IFN–inducible genes in lesional skin (but not in nonlesional skin) of the majority of the psoriatic patients examined in the study; (4) pDCs, the natural IFN-α/β–producing cells are present in lesional skin; and (5) ISG15 and STAT1, 2 type I IFN–inducible proteins, are overexpressed in lesional skin of psoriatic patients.

Of the type I IFN–inducible genes overexpressed in lesional skin of psoriatic patients, STAT1 is a key component in forming the ISGF3 complex. ISGF3 is the heterotrimeric transcriptional factor complex that is formed upon the activation of the type I IFN receptor. It is composed of phosphorylated STAT1, STAT2, and IRF9. Upon translocation to the nucleus, this complex is capable of activating the transcription of type I IFN–inducible genes [28]. IRF7 is the master regulator of the IFN-α/β mediated immune response, and MYD88 and IRF7 govern the induction of CD8^+^ T cell responses [29], [30]. IRF1 functions as a transcriptional activator for the type I IFN–inducible genes [31]. MX1, MX2, and OAS family members OAS1, OAS2, OAS3 mediate resistance to virus infection. ISG15 is a ubiquitin-like protein that becomes conjugated to many cellular proteins upon activation by IFN-α/β [32]. USP18, UBE2L6, and HERC5 are all members of the ISG15 signaling pathways. LAMP3 and CD83 are dendritic cell–marker genes.

Besides the prevalence of overexpression of type I IFN–inducible genes and proteins in lesional skin, we observed leukocyte infiltration (CD4^+^ T cells, CD83^+^ mDCs, and neutrophils; data not shown) in the dermis and epidermis of lesional skin as well as overexpression of genes that are T cell and dendritic cell markers and those that are involved in T cell trafficking. We also showed that mRNAs of TNF-α and TNF-α–inducible genes are overexpressed in lesional skin of psoriatic patients, which may shed light on why anti–TNF-α therapy provides clinical benefit to psoriatic patients. The overexpression of an array of proinflammatory cytokines and/or their inducible genes (TNF-α, IFN-γ, IL-1, IL-8, IL-15, IL-17, IL-19, IL-23, and OSM) in lesional skin suggests that they might be involved in the pathogenesis of the disease.

Type I IFNs may provide the link between innate and adaptive immune responses. They are secreted by pDCs in response to environmental factors like mechanical stress and infections of the skin (2 of the common triggers that cause disease relapse),and drive the activation and expansion of pathogenic T cells leading to psoriasis [24], [26]. Our observations support this link. For example, the presence of pDCs and mDCs and the up-regulation of mRNAs of type I IFN members in lesional skin suggest the overexpression of type I IFN proteins at these sites. Type I IFNs can facilitate activation of mDCs and promote T cell polarization to proinflammatory Th1 effectors [33]. We observed enrichment of mDCs and CD4^+^ T cells, as well as the overexpression of IFN-γ–inducible genes in lesional skin of psoriatic patients. The mRNA for IL-8, a key chemoattractant for neutrophils, and IFN-γ and TNF-α–inducible genes are not overexpressed in nonlesional skin compared with skin from healthy donors. However, all of these genes are overexpressed in lesional skin in a similar manner to the overexpression of mRNAs of type I IFN family members and type I IFN–inducible genes. It remains unclear as to whether IFN-α signaling occurs upstream or downstream of IFN-γ and TNF-α signaling. Ongoing genomic studies for anti IFN-α mAb trial in psoriasis from our laboratory will clarify this issue in the near future.

Type I IFN has been implicated to be involved in the pathogenesis of other autoimmune diseases like systemic lupus erythematosus (SLE), type I diabetes, rheumatoid arthritis, myositis, and Sjögren's syndrome [34]–[36]. The present study demonstrates the utility of analysis of tissues from disease sites using microarray and immunohistochemistry to unveil potential mediators of the pathogenesis of psoriasis. This approach has been successfully used to show the involvement of type I IFNs in the pathogenesis of SLE and myositis [35], [37], [38]. The identification of significant overexpression of mRNA of TNF-α and TNF-α–inducible genes in lesional skin of psoriatic patients and the therapeutic benefit of anti–TNF-α therapies in psoriasis provide further support of using the microarray approach to identify potential therapeutic targets for diseases. The presence of type I IFN–producing pDCs, overexpression of mRNAs of type I IFNs and type I IFN–inducible genes and proteins in lesional skin of psoriatic patients provide a strong rationale for investigating type I IFNs as new therapeutic targets for the treatment of psoriasis.

## Materials and Methods

### Patients and Controls

A total of 21 biopsies from healthy donors (5 samples were obtained from Biochain, 14 samples were from ILSBio, and 2 samples were from Dr. James Krueger, Rockefeller University, New York, NY), 26 paired nonlesional and lesional (all were plaque-type) skin biopsies from 26 psoriatic patients (21 pairs from Asterand, 5 pairs from Dr. Krueger), and 5 lesional skin biopsies from 3 psoriatic patients (all plaque-type) were profiled using the Affymetrix® whole genome array. Of these, 3 nonlesional skin samples either did not yield sufficient cRNA or the scanned arrays had high scaling factors (>2-fold higher than the average) and were excluded from data analysis. All the donors who gave biopsy tissue (both healthy controls and individuals with psoriasis) gave written informed consent for the tissue to be taken and used in this study. Human specimens used in this paper that were procured from both registered commercial vendors and Dr. Krueger at Rockefeller University obtained IRB approval from their respective branches to collect samples.

### Ex vivo stimulation of whole blood from healthy donors

Ex vivo stimulation of whole blood was conducted on blood collected from 3 or 4 donors (MedImmune, Inc.). Blood samples were exposed to treatments of vehicle (1× PBS), a panel of IFN-α subtypes (IFN-α2a, -4b, -5, -6, -7, -8, -10, -16, -17), IFN-β, IFN-ω, IFN-λ, IFN-γ, leukocyte IFN, or TNF-α at concentration of 3xEC_50_. TNF-α and IFN-γ were purchased from R&D Systems (Minneapolis, MN) and the rest of the cytokines were purchased from PBL Biomedical (Piscataway, NJ). Following dosing, the blood was incubated at 37°C, 5% CO_2_ for 4 hours and transferred to a PAXgene RNA tube and inverted 8 to 10 times. The PAXgene tubes were incubated at room temperature for 2 hours and then frozen until processed. All the blood donors gave written informed consent for the blood to be taken and used in this study.

### In vitro stimulation of normal human keratinocytes with type I IFN

Normal human keratinocytes (EpiDerm system, MatTek, Inc.) were grown under serum-free conditions according to manufacturer's instructions and stimulated with either human leukocyte IFN (150 IU/ml, PBL Biomedical Labs), or human IFNα-2a (350 IU/ml, PBL Biomedical Labs). Epidermal cultures were harvested at 4 hours post-treatment for transcript analysis.

### Total RNA extraction and microarray processing

Total RNA was extracted from PAXgene blood and skin biopsies using the PAXgene Blood RNA kit and the Qiagen RNeasy Fibrous Tissue Mini kit (Hilden, Germany), respectively. RNA purity and concentration were determined spectrophotometrically (260/280>1.9). RNA quality was assessed on an Agilent 2100 Bioanalyzer using the RNA 6000 Nano LabChip®.

The generation of biotin-labeled amplified cRNA from 2 µg of total RNA was accomplished with the Affymetrix GeneChip® One-Cycle cDNA Synthesis kit and the Affymetrix GeneChip® IVT Labeling kit. The concentration and purity of the cRNA product were determined spectrophotometrically. Twenty micrograms of each biotin-labeled cRNA was fragmented for hybridization on Affymetrix Human Genome U133 Plus 2.0 GeneChip® arrays. All GeneChip® washing, staining, and scanning procedures were performed with Affymetrix standard equipment. Data capture and initial array quality assessments were performed with the GeneChip Operating Software (GCOS) tool.

### Microarray data analysis

Stratagene's (La Jolla, CA) ArrayAssist® Lite software was used to calculate probe-level summaries (GC-RMA) from the array CEL files. R packages (R Development Core Team) samr and qvalue were used to identify differentially regulated genes. PCA and hierarchical clustering analyses were performed with SpotFire (http://www.spotfire.com/) and R packages. Pathway and network analyses of gene expression data were conducted with the MetaCore™ integrated software suite from GeneGo, Inc. (St. Joseph, MI). The microarray data reported in this manuscript is MIAME-compliant and is available at: http://www.medimmune.com/translationalscience/data/psoriasis2007/.

All authors in the manuscript have access to all data and act as guarantor for the analysis and the manuscript overall.

### Applied Biosystems's TLDA

TLDA from Applied Biosystems was used to determine the fold-change differential for a panel of genes between paired lesional skin and nonlesional skin from psoriatic patients. Genes printed on the array included 9 type I IFN-α subtypes (1, 2, 5, 6, 7, 8, 14, 17, and 21), 3 additional type I IFNs (IFN-β, -κ, -ω), IFN-γ, IFNAR1, IFNAR2, IFNGR1, IFNGR2, and TNF-α. Double-stranded cDNA for each patient sample was preamplified for 10 cycles using the TaqMan PreAmp Master Mix kit (Applied Biosystems). Standard procedures for loading the array were then followed and the array was run on a 7900HT Fast Real-Time PCR System (Applied Biosystems). Data analysis of the resulting Ct values was conducted with SDSv2.2.2 software (Applied Biosystems).

### Fluidigm's Biomark system

#### Preamplification of cDNA and real-time PCR

A mixture of 43 TaqMan Gene Expression assays (Applied Biosystems) was prepared (18S not included) using the TaqMan PreAmp Master Mix Kit (Applied Biosystems). A total of 44 samples were run in triplicate (using 3 different BioMark Real-Time PCR Systems) against a set of 48 TaqMan Gene Expression Assays in BioMark 48.48 Dynamic Array chips (Fluidigm Corp). The normal controls were included on every array. Three identical dynamic arrays were prepared in parallel for each set of 16 samples totaling 9 dynamic arrays. Dynamic arrays were loaded using a NanoFlex 4-IFC Controller (Fluidigm Corp), and real-time reactions were performed and analyzed using BioMark Real-Time PCR System and Analysis software (Fluidigm Corp), respectively. SDs were calculated for each set of 3 replicate reactions and then averaged for each dynamic array. Cts above 20 were excluded from the calculation. Delta-delta Cts (ΔΔCt) were calculated using the mean of the 4 reference genes (GAPDH, TFRC, β2M, and 18S) and a calibrator sample and were converted to fold expression change by the following formula: 2^−ΔΔCt^.

### Immunohistochemistry

One half of each frozen psoriatic, nonlesional, and healthy donor biopsy was embedded in optimum cutting temperature (OCT) compound sectioned at 5 µm, mounted on charged slides (Mercedes Medical, FL), and fixed in cold acetone. Incubation with primary antibodies was performed as follows: BDCA-2 (Miltenyi Biotec, Auburn, CA) at a 1∶5 dilution for 5 hours, STAT1 (Abcam, Cambridge, MA) at a 1∶100 dilution for 4 hours, CD83 (Immunotech, Fullerton, CA) at a 1∶300 dilution for 1.5 hours, ISG15 (Abcam, Cambridge, MA) at a 1∶25 dilution for 2 hours, and CD4 (DakoCytomation, Carpenteria, CA) at a 1∶20 dilution for 1 hour. CD31 (BD Biosciences Pharmingen, San Jose, CA, at 1 µg/mL and incubated for 2 hours) was used as a positive tissue control. The slides were washed and incubated with peroxidase-labeled polymer conjugated to goat anti-mouse immunoglobulin antibody (Envision+; DakoCytomation, Carpenteria, CA) for 30 minutes, then washed with tris-buffered saline (pH 7.2). Detection was performed with 3,3′-diaminobenzidine tetrahydrochloride (DAB+; DakoCytomation) as the chromogen. Slides were counterstained with hematoxylin, dehydrated, and cover slipped.

### Online supplemental material

Figure S1 shows hierarchical clustering of 1384 probe sets differentially regulated by IFN-α subtypes, IFN-β, IFN-γ, and TNF-α in whole blood ex vivo stimulation experiment. Table S1 lists the top 100 probe sets up-regulated in lesional skin compared with nonlesional skin in psoriasis. Table S2 lists the top 50 probe sets down-regulated in lesional skin compared with nonlesional skin in psoriasis. Table S3 lists the top 50 probe sets down-regulated in nonlesional skin compared with skin from healthy donors. Table S4 lists the top 50 probe sets up-regulated from the *in vitro* stimulation of normal human keratinocytes with leukocyte IFN. Online supplemental material is available at http://www.medimmune.com/translationalscience/data/psoriasis2007.

## Supporting Information

Table S1 Fold change (fc; log2 transformed) and q Value (calculated by FDR) of the top 100 probe sets up-regulated in lesional skin compared with nonlesional skin in psoriasis. Also listed are the log2 transformed fold change and q values of these genes when comparing nonlesional skin with normal skin from healthy donors. Type I IFN-inducible genes are designated by the IFNI column. The table is available at: http://www.medimmune.com/translationalscience/data/psoriasis2007/.
(0.04 MB DOC)

Table S2 Fold change (fc; log2 transformed) and q Value (calculated by FDR) of the top 50 probe sets down-regulated in lesional skin compared with nonlesional skin in psoriasis. Also listed are the log2 transformed fold change and q Values of these genes when comparing nonlesional skin with normal skin from healthy donors. All data was GC-RMA normalized. The table is available at: http://www.medimmune.com/translationalscience/data/psoriasis2007/.
(0.03 MB XLS)

Table S3 Fold change (fc; log2 transformed) and q Value (calculated by FDR) of the top 50 probe sets down-regulated in nonlesional skin compared with skin from healthy donors. Also listed are the log2 transformed fold change and q values of these genes when comparing nonlesional skin with lesional skin. All data was GC-RMA normalized. The table is available at: http://www.medimmune.com/translationalscience/data/psoriasis2007/.
(0.03 MB XLS)

Table S4 Fold change (fc; log2 transformed), P values, and mean signal intensities for the top 50 probe sets up-regulated from the in vitro stimulation of normal human keratinocytes with leukocyte IFN. Normal human keratinocytes were stimulated with human leukocyte IFN. Replicates of three were run for both the untreated samples and the stimulated samples. A paired Student's t-test was used to calculate the P values. All data was GC-RMA normalized. The table is available at: http://www.medimmune.com/translationalscience/data/psoriasis2007/.
(0.11 MB XLS)

Figure S1 Hierarchical clustering of 1384 probe sets differentially regulated by IFN-α subtypes and IFN-β (pink), IFN-γ (blue), and TNF-α (brown) in whole blood ex vivo stimulation experiment (see Materials and Methods). Each role corresponds to a single probe set, while each column corresponds to a single sample. Color represents relative expression level of individual probe set as compared with the average expression of the no treatment controls (black). Red represents up-regulation versus control, green down-regulation versus control, while black indicates no change.
(0.50 MB TIF)
