# Correction: Type I Interferon: Potential Therapeutic Target for Psoriasis?

**DOI:** 10.1371/annotation/fbcbcab9-2e87-4ec7-af6e-c6e9e64ad4b3

**Published:** 2009-03-13

**Authors:** Yihong Yao, Laura Richman, Chris Morehouse, Melissa de los Reyes, Brandon W. Higgs, Anmarie Boutrin, Barbara White, Anthony Coyle, James Krueger, Peter A. Kiener, Bahija Jallal

The funding and competing interest statements are incorrect. The funding statement should read: This work was supported by MedImmune. The employees of the funder had a role in study design, data collection and analysis, decision to publish, and preparation of the manuscript.

The competing interests statement should read: All authors except JK are employees of MedImmune.

