# Supplementary figures and images for: Type I Interferon: Potential Therapeutic Target for Psoriasis?
Source: PLoS One. 2008 Jul 16;3(7):e2737. doi: 10.1371/journal.pone.0002737 (PMC2481274; doi:10.1371/journal.pone.0002737)

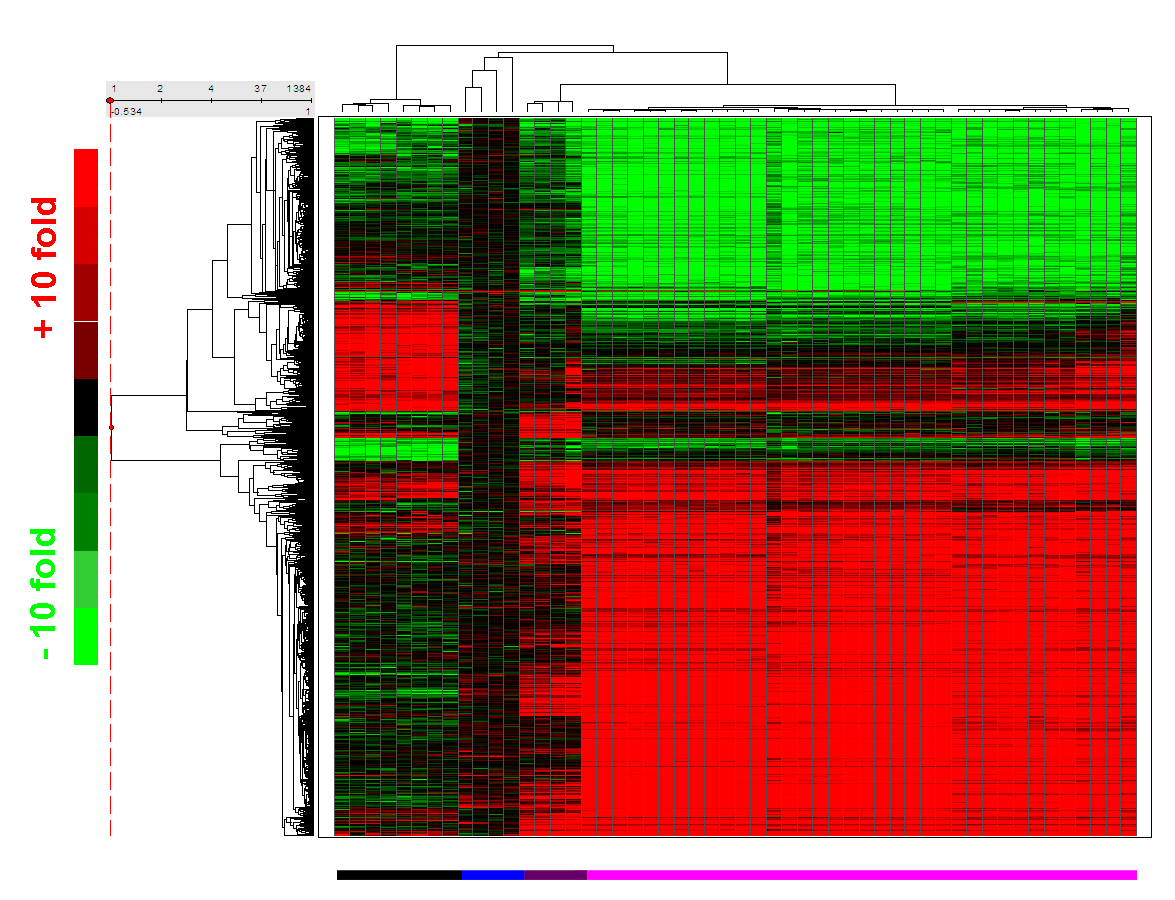

Supplement: Figure S1 — Hierarchical clustering of 1384 probe sets differentially regulated by IFN-α subtypes and IFN-β (pink), IFN-γ (blue), and TNF-α (brown) in whole blood ex vivo stimulation experiment (see Materials and Methods). Each role corresponds to a single probe set, while each column corresponds to a single sample. Color represents relative expression level of individual probe set as compared with the average expression of the no treatment controls (black). Red represents up-regulation versus control, green down-regulation versus control, while black indicates no change. (0.50 MB TIF) [file pone.0002737.s005.tif]
